# Phytoplankton‐derived zwitterionic gonyol and dimethylsulfonioacetate interfere with microbial dimethylsulfoniopropionate sulfur cycling

**DOI:** 10.1002/mbo3.1014

**Published:** 2020-02-29

**Authors:** Björn Gebser, Kathleen Thume, Michael Steinke, Georg Pohnert

**Affiliations:** ^1^ Institute for Inorganic and Analytical Chemistry Friedrich Schiller University Jena Jena Germany; ^2^ School of Life Sciences University of Essex Colchester UK

**Keywords:** dimethylsulfoniopropionate, gonyol, inhibition, metabolism, volatile sulfur species

## Abstract

The marine sulfur cycle is substantially fueled by the phytoplankton osmolyte dimethylsulfoniopropionate (DMSP). This metabolite can be metabolized by bacteria, which results in the emission of the volatile sulfur species methanethiol (MeSH) and the climate‐cooling dimethylsulfide (DMS). It is generally accepted that bacteria contribute significantly to DMSP turnover. We show that the other low molecular weight zwitterionic dimethylsulfonio compounds dimethylsulfonioacetate (DMSA) and gonyol are also widely distributed in phytoplankton and can serve as alternative substrates for volatile production. DMSA was found in 11 of the 16 surveyed phytoplankton species, and gonyol was detected in all haptophytes and dinoflagellates. These prevalent zwitterions are also metabolized by marine bacteria. The patterns of bacterial MeSH and DMS release were dependent on the zwitterions present. Certain bacteria metabolize DMSA and gonyol and release MeSH, in others gonyol inhibited DMS‐producing enzymes. If added in addition to DMSP, gonyol entirely inhibited the formation of volatiles in *Ruegeria pomeroyi*. In contrast, no substantial effect of this compound was observed in the DMSP metabolism of *Halomonas* sp. We argue that the production of DMSA and gonyol and their inhibitory properties on the release of volatiles from DMSP has the potential to modulate planktonic sulfur cycling between species.

## Graphical abstract

Marine sulfur cycling depends to a large extent on the transformation of the dominant algal osmolyte dimethylsulfoniopropionate (DMSP). We show that other low molecular weight zwitterionic dimethylsulfonio (DMS)‐compounds, including dimethylsulfonioacetate (DMSA) and gonyol can serve as alternative substrates fueling the pool of volatile sulfur species from the oceans. We demonstrate that these prevalent zwitterions from algae are metabolized by marine bacteria and that gonyol acts as an inhibitor of bacterial metabolism of the abundant oceanic dimethylsulfoniopropionate. We thus argue that the prevalence of DMSA and gonyol and their inhibitory properties of DMSP‐metabolism modulates the planktonic sulfur cycle and the flux of climate‐active DMS to the atmosphere.

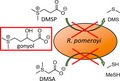

## 1 INTRODUCTION

**Figure 1:**
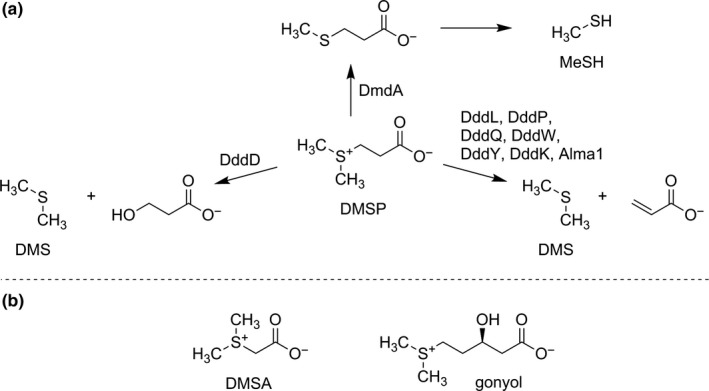
(a), Major catabolic pathways of DMSP. (b), Alternative metabolites containing the dimethylsulfonio structure element found in phytoplankton

Dimethylsulfoniopropionate (DMSP) is a ubiquitous metabolite produced by marine algae and bacteria (Curson et al., 2017; Stefels, 2000). The zwitterionic DMSP fulfills central physiological functions in microalgae as an osmoprotectant (Kirst, 1996), cryoprotectant (Kiene, Linn, & Bruton, 2000; Kirst et al., 1991), and antioxidant (Sunda, Kieber, Kiene, & Huntsman, 2002). An estimated annual production of DMSP of around 10^9^ tons fuels the marine sulfur cycle and it is thus not surprising that marine bacteria and algae have evolved multiple pathways to utilize this resource (Brock et al., 2013; Sievert, Kiene, & Schulz‐Vogt, 2007; Simó, Archer, Pedrós‐Alió, Gilpin, & Stelfox‐Widdicombe, 2002; Vila‐Costa et al., 2006). Marine bacteria can sustain up to 95% of their sulfur and 15% of their carbon requirements through the metabolization of DMSP (Zubkov et al., 2001). Two major metabolic pathways for the degradation of DMSP have been reported from bacteria (Figure 1a). The demethylation/demethiolation pathway initially leads to the formation of 3‐(methylthio) propionate that is the substrate for the release of methanethiol (MeSH) (Taylor & Gilchrist, 1991). The first step of this pathway is encoded in the *dmdA* gene which is widely distributed in marine bacteria (Howard, Sun, Biers, & Moran, 2008; Reisch, Moran, & Whitman, 2008; Varaljay et al., 2012). The DMSP‐cleavage pathways to DMS are catalyzed by several different enzymes forming either acrylate (Alcolombri et al., 2015; Curson, Sullivan, Todd, & Johnston, 2011; Dickschat, Rabe, & Citron, 2015) or 3‐hydroxypropionate as further reaction products (Todd et al., 2007).

Other metabolites that contain the dimethylsulfonio structural element found in DMSP have been identified in marine phytoplankton. This includes dimethylsulfonioacetate (dimethylthetin, DMSA) and gonyol (Figure 1b) (Gebser & Pohnert, 2013; Nakamura, Fujimaki, Sampei, & Murai, 1993; Nakamura et al., 1997). In fact, it is estimated that, depending on the species, up to 10% of the marine DMS may derive from sources other than DMSP (Spielmeyer, Gebser, & Pohnert, 2011a, 2011b). DMSA is recognized by the glycine betaine uptake system in marine bacteria (Kiene, Williams, & Walker, 1998) and can be used for osmoregulatory functions in *Escherichia coli* (Cosquer et al., 1999).

Until recently, dimethylsulfonio compounds besides DMSP were considered to be rather exotic and were reported from only a few algal species (Gebser & Pohnert, 2013; Nakamura et al., 1993, 1997). This view, however, is changing, with new data collected using ultra‐high‐pressure liquid chromatography‐mass spectrometry (UHPLC‐MS) for the direct monitoring of low molecular weight zwitterionic metabolites (Spielmeyer et al., 2011a, 2011b; Spielmeyer & Pohnert, 2010, 2012). Using this methodology, not only the widespread distribution of a diverse family of zwitterionic dimethylsulfonio‐metabolites could be shown but also new and unexpected metabolites, such as dimethylsulfoxoniumpropionate (DMSOP), a biogenic dimethylsulfoxide precursor, were discovered (Thume et al., 2018).

We surveyed the two globally important microalgae *Emiliania huxleyi* and *Prorocentrum minimum* for the regulation of such zwitterionic metabolites during osmoacclimation (Gebser & Pohnert, 2013). Gonyol, previously a metabolite described in the dinoflagellate *Lingulodinium* (*Gonyaulax*) *polyedra* only, was found in both of the tested species and DMSA was detected in *P. minimum* (Gebser & Pohnert, 2013; Nakamura et al., 1993, 1997). This prompted us to undertake a survey of the distribution of these metabolites in a broader screening of phytoplankton species that is presented here. We selected the prominent genetic model species *Phaeodactylum tricornutum* and *Thalassiosira pseudonana,* ecological model species including *Skeletonema costatum* and *E. huxleyi* and dominant key players in plankton blooms such as *Phaeocystis pouchetii* and *Prorocentrom minimum*. Further representatives of the respective phytoplankton classes were chosen to complete the list of investigated species. Indeed, we found that haptophytes and several dinoflagellates produce DMSA and gonyol. We then addressed their function as sources for volatile sulfur species and as mediators of sulfur metabolism in the four marine bacteria, *Ruegeria pomeroyi* DSS‐3, *Halomonas *sp. HTNK1, *Alcaligenes faecalis* M3A, and *Sulfitobacter *sp. EE‐36. These marine bacteria are well known to catabolize DMSP via different DMSP‐dependent DMS‐production pathways involving the central enzymes listed in Table 1.

**Table 1:** Enzymes for DMSP‐dependent DMS production (Ddd) identified in model organisms used in this work

Species	Ddd enzymes	References
*Ruegeria pomeroyi* DSS−3	DddP	(Todd et al., 2009)
	DddQ	(Todd et al., 2011)
	DddW	(Todd *et al.* 2012)
*Sulfitobacter sp.* M3A	DddL	(Curson et al., 2008)
*Alcaligenes faecalis* EE−36	DddY	(Curson, Todd, Sullivan, Johnston, 2011)
*Halomonas sp.* HTNK1	DddD	(Sun et al., 2012)

## 2 MATERIALS AND METHODS

### 2.1 Cultivation of algae

For the quantification of DMSP, DMSA, and gonyol, microalgae were cultured according to a published procedure (Thume et al., 2018). The following strains were utilized and different media were utilized to allow favorable growth conditions for the respective algae (strain numbers refer to the Roscoff Culture Collection [RCC], Belgium Coordinated Organziation of Microorganisms [DCG], Provasoli‐Guillard National Center for Marine Algae and Microbiota [CCMP], Scandinavian Culture Collection for Algae & Protozoa [SCCAP], strains without given number are maintained in our in‐house culture collection and will be made available upon reasonable request). *Prymnesium parvum, S. costatum* RCC75, *Isochrysis galbana*, *Nitzschia* cf. *pellucida* DCG0303, *Navicula* sp. I15, *Phaeodactylum tricornutum* CCMP2561 and SCCAP K‐128, *Stephanopyxis turris*, *Thalassiosira pseudonana* CCMP1335, *T. rotula* RCC841, *T. weissflogii* RCC76, and *Rhodomonas* sp. were cultivated in an artificial seawater medium prepared after Maier and Calenberg (1994). *Phaeocystis pouchetii* AJ01, *Amphidinium carterae* SCCAP K‐0406, and *P. minimum* were cultivated in f/2 medium (Guillard & Ryther, 1962). *Lingulodinium polyedrum* CCAP1221/2 was cultivated in L1 medium (Guillard & Hargraves, 1993). The medium for *E. huxleyi* RCC1217 and RCC1731 was prepared according to (Spielmeyer et al., 2011a, 2011b). Cultivation was initiated from stationary phase stock cultures by a 20‐fold dilution of the cell suspension in 50 ml tissue culture flasks. Before inoculation, the medium was filtered (GF/C grade microfiber filter; GE healthcare) to remove precipitates. All cultures were grown at 12°C (a typical temperature reached in algal blooms in the North Sea (Archer et al., 2002) and North Atlantic (Jickells et al., 2008)) except the arctic isolate *P. pouchetii*, which was kept at 5°C, under a 14:10 light:dark cycle (Osram biolux lamps; 40 µmol/m^2^ s^‐1^ between 400 and 700 nm). Cultures were grown to the exponential phase and then divided into four aliquots of equal volume. These aliquots were 20‐fold diluted with fresh medium and cultivated to the exponential phase before harvesting for extraction.

### 2.2 Algal sample preparation and analysis

Cultures (40 ml) for the screening of dimethylsulfonio‐metabolites were filtered under reduced pressure (GF/C grade microfiber filter; GE healthcare) at 400 mbar, and the filter was immediately transferred to 4 ml glass vials containing 1 ml of methanol for extraction. These samples were vortexed for 1 min before storage at −20°C. For UHPLC‐MS analysis, 50 µl of the extracts was diluted with 90 µl acetonitrile and 10 µl of an aqueous solution of an internal standard mixture (D_6_‐DMSP, D_6_‐DMSA, and D_3_‐gonyol, the concentration of the standards was between 0.1 and 300 µM dependent on the concentration of the analytes that was estimated in a first prescreening). For quantification of unmetabolized substrates immediately after quantification of DMS and MeSH, 100 µl of bacteria culture inoculations was diluted with 100 µl methanol. These suspensions were stored at −20°C until further analysis. For the gonyol‐dependent DMSP‐metabolization experiment, aliquots of 500 µl bacteria culture were added to 500 µl methanol in a microcentrifuge tube. After the addition of the isotope‐labeled internal standards (D_6_‐DMSP, D_6_‐DMSA, and D_3_‐gonyol) samples were centrifuged for 5 min at 16,100 *g*, and the supernatant was frozen at −20°C until measurement. An aliquot of the suspensions (50 µl) was diluted with 200 µl acetonitrile/water 9:1 in microcentrifuge tubes.

All samples were centrifuged before the measurement of the supernatant (5 min, 4,500 *g*). The supernatant was directly injected into an Acquity UHPLC (Waters) equipped with a SeQuant ZIC^®^‐HILIC column (5 μm, 2.1 × 150 mm, SeQuant, Umeå). A Q‐ToF micro mass spectrometer (Waters Micromass) with electrospray ionization in positive ionization mode was used for detection and identification. For separation and quantification, the method of Gebser and Pohnert (2013) was used. The eluent consisted of 2% acetonitrile and 0.1% formic acid in high purity water (solvent A) and 90% acetonitrile with 10% water and 5 mM ammonium acetate (solvent B). The flow rate was set to 0.60 ml/min. The separation was performed at 35°C according to (Spielmeyer et al., 2011a, 2011b).

For quantification of zwitterionic substances, relative response factors were determined by the measurement of an equimolar mixture of the analyte and the corresponding isotopically labeled internal standard. Response factors were calculated by comparison of the peak area of the analytes with the peak area of the corresponding internal standard.

### 2.3 Cultivation of bacteria

Stock cultures of *R. pomeroyi* DSS‐3 and *Sulfitobacter *sp. EE‐36 were grown in marine basal medium MBM (Baumann & Baumann, 1981). *Alcaligenes faecalis* M3A and *Halomonas *sp. HTNK1 were cultivated in M9 minimal medium (Sigma‐Aldrich). All cultures were grown under gentle shaking at 28°C with the addition of 10 mM sodium succinate as a carbon source. Exponentially growing cultures were selected for experiments on substrate utilization.

### 2.4 Incubation of bacteria with zwitterionic molecules

Prior to incubation, 3 ml of bacteria cultures were washed three times by centrifugation (5 min at 16,100*g*) and resuspension in succinate‐free medium to remove any excess organic carbon. For incubation experiments, all bacteria cultures were diluted with succinate‐free medium to the same optical density (OD_600_) of 0.1. Aliquots (450 µl) of these cultures were transferred into autoclaved 5 ml screw‐cap glass vials with PTFE/silicone septa. Medium without the addition of bacteria was used as control. After the addition of aqueous solutions of the substrates to a final concentration of 3.3 µM, the vials were sealed and placed on a heated shaker at 28°C for 24 hr. Additionally, mixtures of the respective substrates DMSP/DMSA, DMSP/gonyol, DMSA/gonyol, and DMSP/DMSA/gonyol (final concentration of each substrate 3.3 µM) were applied. Controls were investigated without the addition of substrates. Four biological replicates were prepared for each treatment and control. After 24 hr of incubation, methanethiol (MeSH) and dimethylsulfide (DMS) were quantified using headspace sampling and direct injection into a GC‐FPD system (see below).

### 2.5 Stability of gonyol at alkaline pH

Gonyol (3.3 µM) in 1N NaOH was incubated for 1 hr at 30°C before the quantification of volatiles. Samples were vigorously shaken several times during incubation and prior to the measurements to achieve equilibrium between the liquid and gas phases. Headspace analysis was performed with GC‐FPD as outlined below.

### 2.6 GC‐FPD measurement of MeSH and DMS

For quantification of MeSH and DMS in the headspace of the samples, the sealed vials were flushed with oxygen‐free nitrogen for 1 min at a flow rate of 60 ml/min and cryogenic enrichment of the samples was carried out according to Franchini and Steinke (2017). After rapid heating of the sample loop using freshly boiled water, the samples were introduced to a gas chromatograph (GC‐2010, Shimadzu) equipped with a 30 m × 0.53 mm × 5 µm HP‐1 capillary column (Agilent) and a flame‐photometric detector. The GC oven was set isothermally at 40°C with helium as carrier gas at a flow rate of 10.56 ml/min. The flame gases for the FPD, compressed air and hydrogen, were set to 70 and 60 ml/min, respectively. Calibration for DMS was done by pipetting aqueous DMSP standard solutions to 450 µl 1M NaOH in a 4.92 ml screw‐cap vial with PTFE/silicone septa to give final concentrations of 0.03, 0.33, 0.66, 1.00, 1.30, 1.70, and 2.00 µM. The vials were sealed immediately after the addition of the DMSP standard. After incubation for 24 hr at 30°C, the samples were analyzed as outlined above. For MeSH calibration, 10.9 mg sodium methanethiolate (Sigma‐Aldrich) was dissolved in 1 ml 10M NaOH as a stock solution. Dilutions of the stock solution were prepared in 1M NaOH. This standard solution was added to 450 µl 2M sulfuric acid in sealed 5 ml screw‐cap vials to give final concentrations of 0.03, 0.06, 0.33, 0.65, 0.96, 1.32, 1.65, and 2.00 µM MeSH. The samples were immediately sealed, incubated, and analyzed as described above.

### 2.7 Quantification of bacterial growth

To determine the effect of the substrates on bacterial growth, stock cultures were cultivated and washed as outlined above. Bacteria cultures were transferred to autoclaved 20 ml headspace vials with cotton stoppers for further cultivation (28°C, shaking). After addition of 3.3 µM DMSP, DMSA, and gonyol, respectively, bacterial growth was monitored for 72 hr by measuring the optical density at 600 nm (OD_600_) in standard single‐use polystyrene cuvettes (Sarstedt AG & Co.) using a two‐beam UV‐Vis spectrophotometer (Specord M42, Carl Zeiss Jena). For each strain, a control without substrate addition was prepared. Measurements were performed with three biological replicates.

### 2.8 Bacterial consumption of MeSH and DMS

In order to determine the consumption of volatile DMS and MeSH by the investigated bacteria species, cultures were prepared as mentioned above (5 ml screw‐cap vial, 450 µl culture, OD_600_ = 0.1). To each culture, freshly prepared aqueous solutions of DMS (Sigma‐Aldrich) or MeSH (Fisher Scientific) were added as a substrate to reach a final concentration of 3.3 µM. Vials were sealed immediately after addition of the substrate solution, and samples were incubated for 24 hr at 28°C under continuous shaking. Control treatments included MBM and M9 media. Due to the high reactivity of MeSH, we determined possible unspecific interactions with bacteria samples as an additional control. Therefore, samples were boiled for 2 min after measurements for bacterial consumption of MeSH. This treatment stopped bacterial metabolism and removed the remaining MeSH. After cooling to room temperature, an aqueous solution of MeSH was added as mentioned above and the samples were incubated again for 24 hr at 28°C. After incubation, 25 µl of the gas phase was taken from the headspace of the samples with a gastight syringe (Hamilton) and injected into a GC‐MS system (Thermo Finigan, ISQ) equipped with a Zebron ZB‐1ms column (60 m × 0.25 mm × 1 µm, Phenomenex). The oven temperature was set to 50°C. DMS and MeSH in the gas phase were determined by integrating the peak areas of the corresponding peaks in the mass traces, *m*/*z* = 62 and *m*/*z* = 48, respectively. Measurements were performed with five biological replicates.

## 3 RESULTS AND DISCUSSION

### 3.1 Distribution of dimethylsulfonio‐metabolites

We selected 16 phytoplankton species (eight diatoms, three dinoflagellates, a cryptophyte, and four haptophytes) to screen for intracellular DMSA and gonyol concentrations. As reported previously, gonyol was abundant in *L. polyedrum*, the dinoflagellate from which it was initially isolated (Gebser & Pohnert, 2013; Nakamura et al., 1993, 1997), with concentrations exceeding that of DMSP by more than 10‐fold. In addition, our screening revealed this metabolite in quantities relative to the abundance of DMSP of ca. 5% in the haptophytes *E. huxleyi* and *Isocrysis galbana* and up to 24% in the dinoflagellates (Table 2). Gonyol was below the detection limit in any of the diatoms investigated.

**Table 2:** Abundance of DMSA and gonyol in different phytoplankton cultures, (n.d.) not detected. Values in brackets represent the standard deviation (*n* = 4, *I. galbana* *n* = 3)

Species	Taxonomic group	Strain no.	DMSA [fmol/cell]	gonyol [fmol/cell]	DMSP [fmol/cell]
*Navicula *sp.	diatom	I15	*n*.d.	*n*.d.	0.0075 (0.0017)
*Nitzschia *cf *pellucida*	diatom	DCG303	*n*.d.	*n*.d.	0.11 (0.0423)
*Phaeodactylum tricornutum*	diatom	CCMP2561	0.0192 (0.0012)	*n*.d.	0.528 (0.067)
*Phaeodactylum tricornutum*	diatom	SCCAP K−1280	0.0125 (0.0024)	*n*.d.	1.32 (0.15)
*Skeletonema costatum*	diatom	RCC75	0.0027 (0.0013)	*n*.d.	6.56 (2.06)
*Stephanopyxis turris*	diatom		0.191 (0.107)	*n*.d.	4.63 (1.9)
*Thalassiosira weissflogii*	diatom	RCC76	*n*.d.	*n*.d.	0.848 (0.05)
*Thalassiosira rotula*	diatom	RCC841	0.0204 (0.0038)	*n*.d.	2.4 (0.89)
*Thalassiosira pseudonana*	diatom	CCMP1335	0.0139 (0.0009)	*n*.d.	1.22 (0.16)
*Rhodomonas *sp.	cryptophyte		9.73 (0.22)	*n*.d.	0.116 (0.028)
*Isochrysis galbana*	haptophyte		0.0054 (0.0006)	0.256 (0.02)	4.69 (0.27)
*Emiliania huxleyi*	haptophyte	RCC1731	0.0005 (0.0001)	0.11 (0.013)	4.44 (0.406)
*Emiliania huxleyi*	haptophyte	RCC1217	0.0013 (0.0003)	0.176 (0.026)	4.83 (0.57)
*Prymnesium parvum*	haptophyte		0.0099 (0.0018)	0.255 (0.08)	16.2 (4.36)
*Phaeocystis pouchetii*	haptophyte		*n*.d.	0.063 (0.017)	4.63 (0.59)
*Prorocentrum minimum*	dinoflagellate		7.66 (0.91)	17.08 (3.48)	304.4 (61.2)
*Amphidinium carterae*	dinoflagellate	SCCAP K−0406	0.0116 (0.0123)	3.23 (0.75)	109.1 (27.2)
*Lingulodinium polyedrum*	dinoflagellate	CCMP1121/2	< 0.15	298.9 (40.5)	25.2 (4.7)

Most species also contained DMSA (Table 2). In the cryptophyte *Rhodomonas *sp., DMSA exceeded the amount of DMSP. In other species, the amount ranged from 0.01% (*A. carterae*) to 4% (*S. turris*) relative to the abundance of DMSP. Given the few reports of gonyol and DMSA in the literature, their universal distribution and sometimes high concentrations are rather surprising. It can be concluded that our understanding of the diversity and distribution of dimethylsulfonio compounds and their metabolic pathways is incomplete due to the methodological limitations of many previous studies. The predominantly used analytical procedures for the indirect quantification of DMSP rely on its conversion to DMS during chemical hydrolysis with strong base and detection of the released DMS (Kiene, 1992; Malin, Turner, Liss, Holligan, & Harbour, 1993; van Rijssel & Gieskes, 2002; Vogt, Rabenstein, Rethmeier, & Fischer, 1998). This indirect test fails to distinguish between DMS from DMSP or several other dimethylsulfonio precursors after alkaline hydrolysis. Gonyol and DMSA did not release substantial amounts of DMS under alkaline conditions with 1.9 ± 0.1% of the initially applied gonyol detected as DMS after 1 hr in 1 M NaOH at 30°C. This low DMS release can be ascribed to impurities remaining from the chemical synthesis of gonyol and the lack of reactivity in alkaline solution can be explained by the fact that base‐mediated DMSP lysis requires abstraction of the acidic proton in α‐position relative to the acid group but that gonyol lysis would require attack on the γ‐proton that is not acidic. In contrast, the method used in this study allows the direct quantification of a multitude of low molecular weight zwitterionic metabolites including DMSP, DMSA, and gonyol but also of related nitrogen‐containing metabolites such as the DMSP‐analogue glycine betaine (Spielmeyer et al., 2011a, 2011b; Spielmeyer & Pohnert, 2012).

### 3.2 Metabolization of dimethylsulfonio‐metabolites

As a consequence of this broad distribution of DMSP, DMSA, and gonyol, the question arises on how these compounds influence and contribute to the marine microbial sulfur cycle. Laboratory experiments that challenged bacteria with pure DMSP showed a significant turnover of this compound (Gonzalez, Kiene, & Moran, 1999; Kiene, Linn, Gonzalez, Moran, & Bruton, 1999; Zubkov et al., 2001, 2002). Results in Table 2 suggest that these experiments might have been oversimplified, since bacteria will be frequently exposed to a complex mixture of dimethylsulfonio‐metabolites including DMSP, DMSA, and gonyol and not to the single compound. To characterize the bacterial utilization and degradation of these zwitterionic osmolytes, we chose the four well‐studied model species *R. pomeroyi* DSS‐3, *Halomonas *sp*.* HTNK1, *A. faecalis* M3A, and *Sulfitobacter *sp*.* EE‐36 for which information about DMSP‐cleavage activities, corresponding genes and the demethylation/demethiolation pathway are available (Table 1), (Curson, Rogers, Todd, Brearley, & Johnston, 2008; Curson, Sullivan, et al., 2011; Desouza & Yoch, 1995; Miller & Belas, 2004; Sun, Curson, Todd, & Johnston, 2012; Todd, Curson, Dupont, Nicholson, & Johnston, 2009; Todd et al., 2010).

The metabolism of the individually added substrates DMSP, DMSA, and gonyol by bacteria was calculated as the percentage of the initial substrate concentration (3.3 µM) remaining after 24 hr incubation. The applied substrate concentration is high compared with the batch availability of these compounds in natural seawater, which is typically in the low nanomolar range for DMSP (Kiene & Slezak, 2006). However, local concentrations surrounding intact phytoplankton cells and generated during senescence will provide microenvironments with comparably high concentrations of the respective metabolites (Grosser et al., 2012; Seymour, Amin, Raina, & Stocker, 2017). Phytoplankton‐associated bacteria are therefore exposed to high concentrations of organic metabolites which include DMSP, DMSA, and gonyol and our findings describe processes associated with such microenvironments.

**Figure 2:**
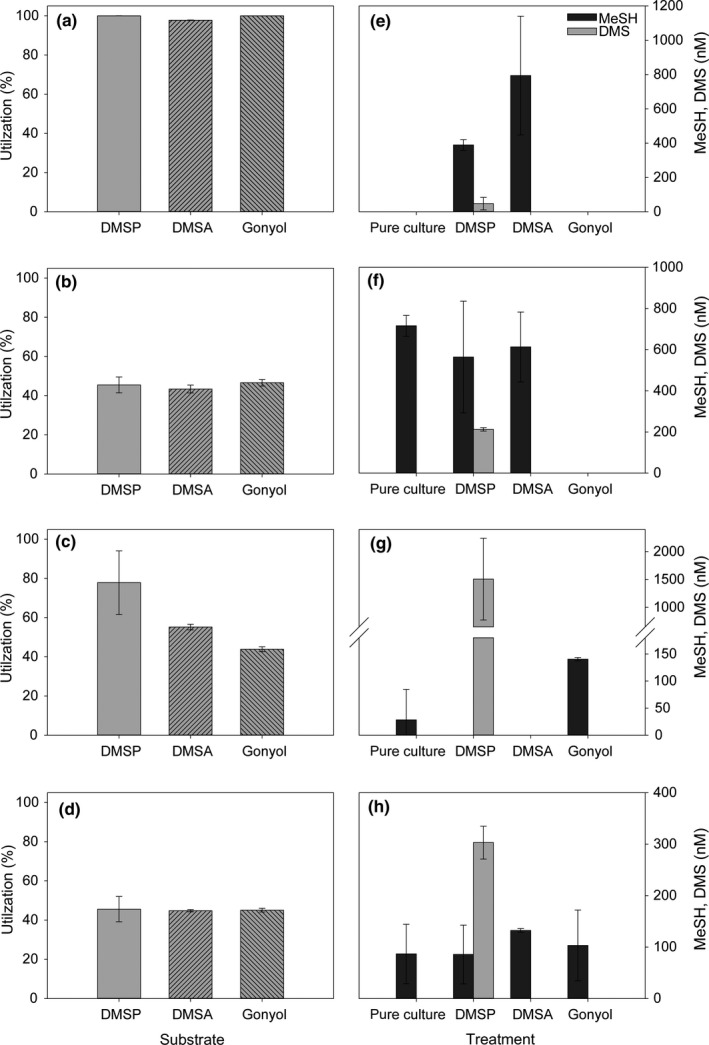
Mean utilization (in %) of the different substrates and concentrations of volatile sulfur compounds methanethiol (MeSH) and dimethylsulfide (DMS) released by *R. pomeroyi* DSS‐3 (a, e), *Sulfitobacter *sp. EE‐36 (b, f), *A. faecalis* M3A (c, g) and *Halomonas *sp. HTNK1 (d, h). Measurements were performed after 24 hr incubation, volatiles are given as net accumulated concentrations over 24 hr in sealed tubes. Error bars represent the standard deviation between biological replicates, *n* = 4. Control measurements of bacteria cultures with the addition of 10 mM sodium succinate as a carbon source are referred to as pure culture. Statistical evaluation is given in Table 3

Quantifying DMSP, DMSA, and gonyol utilization reveals the overall metabolic transformation of these substrates regardless of the degradation or conjugation pathways. It is remarkable that *R. pomeroyi* quantitatively utilized all three added substrates (Figure 2a). The added gonyol was synthesized as a racemic mixture and both enantiomers are metabolized. The pathway for gonyol metabolism is thus not enantioselective or different pathways for both enantiomers are involved. All other bacteria also accepted the three substrates, but did not metabolize them quantitatively during the incubation time. DMSP, DMSA, and gonyol were utilized with similar efficiency in *Sulfitobacter* sp. and *Halomonas* sp. (Figure 2b, d). Both bacteria metabolized ca. 45% of the initially supplied substrates within 24 hr of incubation. *Alcaligenes faecalis* showed a significantly faster transformation of DMSP (77.8 ± 16.2%) compared with DMSA (55.2 ± 1.4%, *t*‐test: *p* = .029, *n* = 4) and gonyol (43.9 ± 1.2%, *p* = .029), respectively (Figure 2c).

**Table 3:** Statistical analyses ‐ *p* values indicate a statistical difference between treatment a and treatment b in the specific bacterial culture, *n* = 4 independent biological replicates

a: *p* values ‐ Figure 2a‐d Utilization					
Treatment a	Treatment b	*R. pomeroyi*	*A. faecalis*	*Sulfitobacter* sp.	*Halomonas* sp.
DMSP	DMSA	<0.001	0.032	0.387	0.811
DMSP	gonyol	0.53	0.006	0.624	0.877
DMSA	gonyol	<0.001	<0.001	0.048	0.622

b: *p* values ‐ Figure 2f‐H MeSH/DMS release									
Treatment a	Treatment b	*R. pomeroyi*		*A. faecalis*		*Sulfitobacter* sp.		*Halomonas* sp.	
		MeSH	DMS	MeSH	DMS	MeSH	DMS	MeSH	DMS
Pure	DMSP	<0.001	0.040	0.356	0.006	0.314	<0.001	0.981	<0.001
Pure	DMSA	0.004	1.000	0.356	1.000	0.290	1.000	0.164	1.000
Pure	gonyol	1.000	1.000	0.007	1.000	<0.001	1.000	0.725	1.000
DMSP	DMSA	0.059	0.040	1.000	0.006	0.770	1.000	0.153	<0.001
DMSP	gonyol	<0.001	0.040	<0.001	0.006	0.006	<0.001	0.708	<0.001
DMSA	gonyol	0.004	1.000	<0.001	1.000	<0.001	1.000	0.428	1.000

c: *p* values ‐ Figures 2 and 3 MeSH/DMS release									
Treatment a	Treatment b	*R. pomeroyi*		*A. faecalis*		*Sulfitobacter* sp.		*Halomonas* sp.	
		MeSH	DMS	MeSH	DMS	MeSH	DMS	MeSH	DMS
Pure	DMSP + DMSA	0.045	0.034	0.0.356	<0.001	0.716	<0.001	0.988	<0.001
Pure	DMSP + gonyol	1.000	1.000	0.002	<0.001	0.012	<0.001	0.188	<0.001
Pure	DMSA + gonyol	1.000	1.000	0.003	1.000	0.217	1.000	0.906	1.000
Pure	DMSP DMSA + gonyol	1.000	1.000	0.011	<0.001	0.085	<0.001	0.201	<0.001
DMSP	DMSP + DMSA	0.321	0.117	1.000	0.004	0.442	<0.001	0.994	0.002
DMSP	DMSP + gonyol	<0.001	0.040	<0.001	0.343	0.174	0.062	0.192	0.009
DMSP	DMSA + gonyol	<0.001	0.040	<0.001	0.006	0.917	<0.001	0.888	<0.001
DMSP	DMSP + DMSA +gonyol	<0.001	0.040	0.003	0.007	0.468	0.763	0.187	0.730
DMSA	DMSP + DMSA	0.043	0.034	1.000	<0.001	0.511	<0.001	0.157	<0.001
DMSA	DMSP + gonyol	0.004	1.000	<0.001	<0.001	0.065	<0.001	0.009	<0.001
DMSA	DMSA + gonyol	0.004	1.000	<0.001	1.000	0.814	1.000	0.233	1.000
DMSA	DMSP + DMSA +gonyol	0.004	1.000	0.003	<0.001	0.274	<0.001	0.108	<0.001
Gonyol	DMSP + DMSA	0.045	0.034	<0.001	<0.001	<0.001	<0.001	0.714	<0.001
Gonyol	DMSP + gonyol	1.000	1.000	0.018	<0.001	0.049	<0.001	0.136	<0.001
Gonyol	DMSA + gonyol	1.000	1.000	0.026	1.000	<0.001	1.000	0.813	1.000
Gonyol	DMSP + DMSA +gonyol	1.000	1.000	0.109	<0.001	0.031	<0.001	0.496	<0.001
DMSP + DMSA	DMSP + gonyol	0.045	0.034	<0.001	0.005	0.025	<0.001	0.191	0.144
DMSP + DMSA	DMSA + gonyol	0.045	0.034	<0.001	<0.001	0.390	<0.001	0.894	<0.001
DMSP + DMSA	DMSP + DMSA +gonyol	0.045	0.034	0.003	0.164	0.133	<0.001	0.192	<0.001
DMSP + gonyol	DMSA + gonyol	1.000	1.000	0.457	<0.001	0.097	<0.001	0.169	<0.001
DMSP + gonyol	DMSP + DMSA +gonyol	1.000	1.000	0.708	0.009	0.541	0.217	0.011	<0.001
DMSA + gonyol	DMSP + DMSA +gonyol	1.000	1.000	0.438	<0.001	0.359	<0.001	0.280	<0.001

Note

For comparison of two groups, an unpaired two‐tailed *t*‐test was performed. All statistical analyses were performed with a 95% confidence interval using Sigma‐Plot version 13.0. *p* > .05 is considered not significantly different.

These results demonstrate that the widely distributed zwitterionic metabolites DMSA and gonyol might represent additional carbon and energy sources since they can be readily metabolized by marine bacterioplankton. Although not leading to the release of DMS, the catabolism of DMSA and gonyol results in the production of MeSH.

### 3.3 Release of MeSH and DMS from DMSP, gonyol, and DMSA

Monitoring the release of volatiles during the metabolization of the substrates allowed deducing if enzymes for DMSP‐dependent DMS‐production or demethylation/demethiolation pathways were involved in the respective transformations. The first pathway would result in DMS release while MeSH is produced by demethylation/demethiolation. Since we observed that DMSP, DMSA, and gonyol were metabolized by all investigated bacteria, we aimed to characterize the pathways involved by quantifying the volatile sulfur‐metabolites MeSH and DMS. The release of these volatiles from synthetic DMSP, DMSA, and gonyol was determined by headspace analysis and GC/FPD measurements in bacterial cultures which were not preacclimated to the utilization of these substrates. Control measurements of the substrates in medium revealed that neither DMSP, DMSA nor gonyol released any of these volatiles in the absence of bacteria (data not shown). Therefore, DMS and MeSH release resulted from the intrinsic enzymatic activity in the examined bacteria (Figure 2). Apart from *R. pomeroyi*, the tested bacteria produced MeSH even without incubation with any of the substrates. This might be caused by assimilatory sulfate reduction and metabolism of resulting sulfur‐containing metabolites produced by the bacteria.

All bacteria tested converted DMSP to DMS, but with different efficiencies. While DMS concentration after 24 hr of incubation of *R. pomeroyi* with DMSP was 47 ± 36 nM (Figure 2e), *A. faecalis* showed a 32‐fold higher DMS release of 1,510 ± 730 nM (Figure 2g). *Sulfitobacter* sp. and *Halomonas* sp. released around 200–300 nM of DMS. These findings are consistent with experiments by Todd et al. (2011) and Curson, Sullivan, et al. (2011) who calculated similar DMSP‐to‐DMS conversions in *R. pomeroyi* and *A. faecalis*. Our data regarding DMSP‐dependent DMS production are likely underestimates of the full metabolic potential since bacteria were not pre‐exposed to DMSP and, therefore, were not acclimated to utilize this substrate. Other studies on DMSP consumption in bacteria report that cultures that were grown on high concentrations (5 mM) of DMSP maximize the expression of enzymes required for DMSP catabolism (Curson, Sullivan, et al., 2011; Desouza & Yoch, 1995; Todd et al., 2011). We could not demonstrate DMS release from substrates other than DMSP (Figure 2e‐H). This indicates that enzymes involved in DMS production in these bacteria are substrate‐specific for DMSP. This high specificity might be explained by the enzyme mechanism recently identified for the DMSP lyase DddQ, from *R. lacuscaerulensis* (Li et al., 2014). This lyase relies on the abstraction of an acidic alpha proton from DMSP resulting in concomitant beta‐elimination of DMS and the release of acrylate. This elimination mechanism is excluded for the shorter chain length homolog DMSA and the longer chain length homolog gonyol due to the lack of an acidic proton in a suitable position of the substrate to support DMS elimination.

In contrast to the pathways leading to DMS, substrate utilization via the demethylation/demethiolation pathway is apparently not limited to DMSP. We detected elevated MeSH release after incubation of *R. pomeroyi* with DMSA (790 ± 350 nM MeSH; Figure 2e). MeSH release from DMSP is described in several bacteria (Miller & Belas, 2004; Taylor & Gilchrist, 1991), and here we extend this metabolic activity to the substrate DMSA. *Ruegeria pomeroyi* also releases MeSH from DMSP but the involved enzymes might not be the same since the electronic situation in both substrates is entirely different. While in DMSP enzymatic abstraction of the acidic α‐proton facilitates its lysis, this is not possible for DMSA. In this metabolite demethylation and demethiolation by the attack on the C2‐position would represent a plausible pathway for MeSH release. This is supported by findings of Reisch et al. (2008) who show that the enzyme DmdA that catalyzes the first reaction step of the demethylation/demethiolation pathway of DMSP does not recognize DMSA. An additional demethylation/demethiolation pathway in bacteria that accepts DMSA as a substrate might thus be responsible for the observed volatile production. Interestingly, this alternative pathway is efficient; MeSH release from DMSA in *R. pomeroyi* (790 ± 350 nM) was higher than the demethylation/demethiolation activity for DMSP that accounted for only 390 ± 31 nM MeSH release (Figure 2e). Due to a lower outlier in the DMSA measurements (Dean‐Dixon test, *N* = 4, *α* = .1), the difference is not significant (*p* = .343, without outlier: *p* ≤ .001). The importance of this newly identified source for MeSH production lies in the high relevance of MeSH for sulfur assimilation by marine bacteria (Kiene et al., 1999; Visscher, Taylor, & Kiene, 1995). In the other three bacteria tested DMSA is not metabolized to any of the two volatiles since their concentrations are not exceeding those in the control or since they are not produced at all. In *A. faecalis,* MeSH release might be inhibited in the presence of DMSA (Figure 2g).

Even if all bacteria metabolized gonyol, no DMS or MeSH was released from this substrate in *R. pomeroyi*, *Halomonas* sp., and *Sulfitobacter* sp. (Figure 2e, f). This indicates the involvement of an alternative pathway that does not lead to cleavage of the C5‐S bond. As discussed above for the base‐mediated transformation of gonyol, the lack of an acidic γ‐proton does not allow a DMSP‐lyase type pathway (Alcolombri et al., 2015). *Alcaligenes faecalis* responds to gonyol with a significantly higher MeSH release (140 ± 3 nM) compared to the untreated control (*p* = .029) (Figure 2g). It remains unclear whether this can be ascribed to a higher demethylation/demethiolation activity or a decrease in MeSH metabolism.

**Figure A1:**
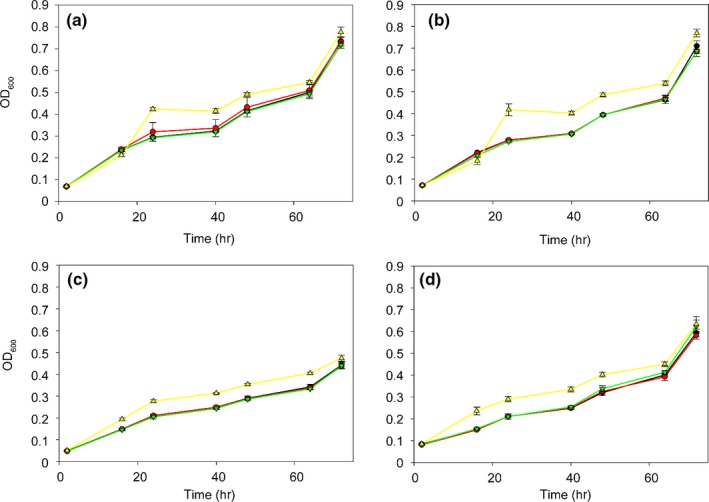
Growth curves of *R. pomeroyi* DSS‐3 (a), *Sulfitobacter* sp. (b), *A. faecalis* (c), and *Halomonas* sp. (d) over 72 hr at 28°C under constant agitation. Black lines show controls where bacteria were cultivated in MBM (a, b) and M9 minimal medium with 10 mM sodium succinate as carbon source (c, d) (●). Colored lines show the growth after the addition of the alternative carbon sources: Colored lines show the growth after the addition of 3.33 μM DMSP (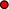), 3.33 μM DMSA (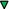), and 3.33 μM gonyol (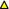). Error bars represent the standard deviation of biological replicates (*n* = 3)

Interestingly, gonyol inhibited the MeSH release in *Sulfitobacter *sp. (Figure 2f) (*p* = .029 in comparison to the control). In contrast, MeSH release was not affected by the addition of DMSP (*p* = .314) or DMSA (*p* = .290). A possible antibacterial function of gonyol which could explain this result can be excluded since a disk‐diffusion test with *Sulfitobacter* sp. and different gonyol concentrations was negative, and growth was not inhibited in the presence and absence of this substrate (Figure A1 in Appendix 2). In fact, gonyol slightly increased bacterial optical density in comparison to the control as well as to the DMSP and DMSA treatments.

**Figure A2:**
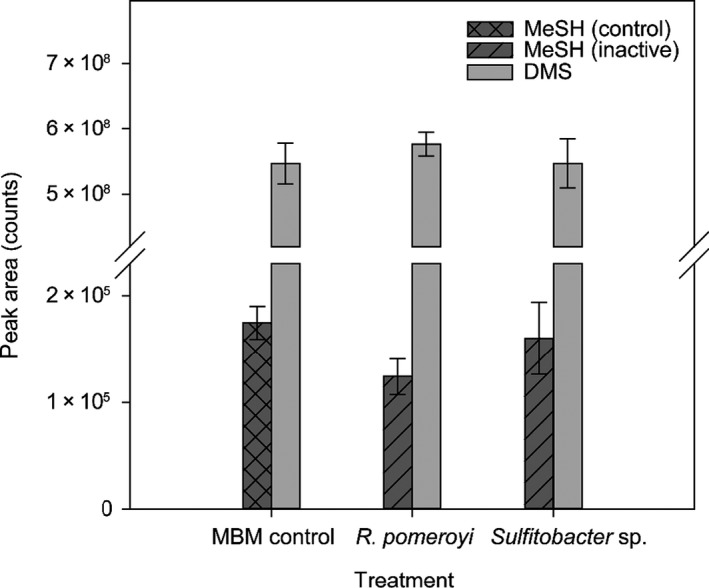
Bacterial consumption of DMS and MeSH, respectively, after 24 hr at 28°C under constant agitation. Bars represent the peak area of the corresponding mass traces of MeSH (*m*/*z* = 48) and DMS (*m*/*z* = 62). MBM control corresponds to the noninoculated marine basal medium. Inactive treatment refers to cultures that were boiled for inactivation of enzyme activity. In the active cultures (living bacteria), no MeSH could be detected after 24 hr incubation Error bars represent the standard deviation of individual replicates (*n* = 5)

**Figure A3:**
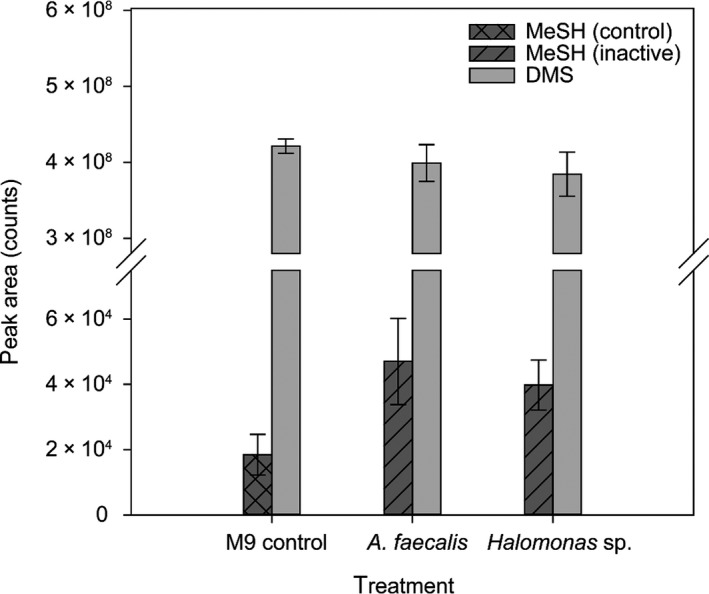
Bacterial consumption of DMS and MeSH, respectively, over 24 hr at 28°C under constant agitation. Columns represent the peak area of the corresponding mass traces of MeSH (*m*/*z* = 48) and DMS (*m*/*z* = 62), respectively. M9 control corresponds to a noninoculated M9 minimal medium. Inactive treatment refers to cultures that were boiled for inactivation of enzyme activity. In the active cultures (living bacteria), no MeSH could be detected after 24 hr incubation. Error bars represent the standard deviation of individual replicates (*n* = 5)

In all investigated cases, apart from the DMSP catabolism in *A. faecalis*, the release of volatile sulfur compounds explained only a minor fraction of the overall transformed substrates. It is thus obvious that the bacteria utilize sulfur and presumably carbon of all administered substrates via hitherto unidentified pathways and that volatile emission represents only a side route. Bacteria have been shown to consume the volatiles DMS and MeSH (Kiene et al., 1999; Reisch, Moran, & Whitman, 2011) so the concentrations of these metabolites can be dependent on enzymatic production and consumption. To assess DMS and MeSH consumption, we conducted short‐term (24 hr) incubations with these gases and quantified their concentrations in the absence (medium control) and presence of bacteria using GC‐MS. There was no significant difference in DMS concentration between *R. pomeroyi*, *Sulfitobacter* sp., *A. faecalis* and the corresponding medium controls (Figures A2 and A3 in Appendix 2). This indicates that DMS was not metabolized in significant amounts so that gross consumption was negligible. *Halomonas* sp. showed 8% consumption of DMS within 24 hr compared to the control M9 medium. As a consequence, DMS concentrations determined in our study should be regarded as a good approximation for net production over the incubation period. In contrast, MeSH as a substrate is metabolized by all bacteria. Inactivated bacteria (boiled controls) showed comparable concentrations as the medium controls so that nonspecific loss of the reactive MeSH in the presence of organic material can be ruled out (Figures A2 and A3 in Appendix 2). This suggests that the detected net production of MeSH in our experiments underestimates the gross production rate resulting from the close coupling of production and consumption processes.

In certain combinations (gonyol with *R. pomeroyi* and *Sulfitobacter* sp. or DMSA with *A. faecalis*), no net volatile emission was observed despite substantial metabolization of the administered substrates.

### 3.4 Inhibition of volatile release from DMSP by gonyol and DMSA

**Figure 3:**
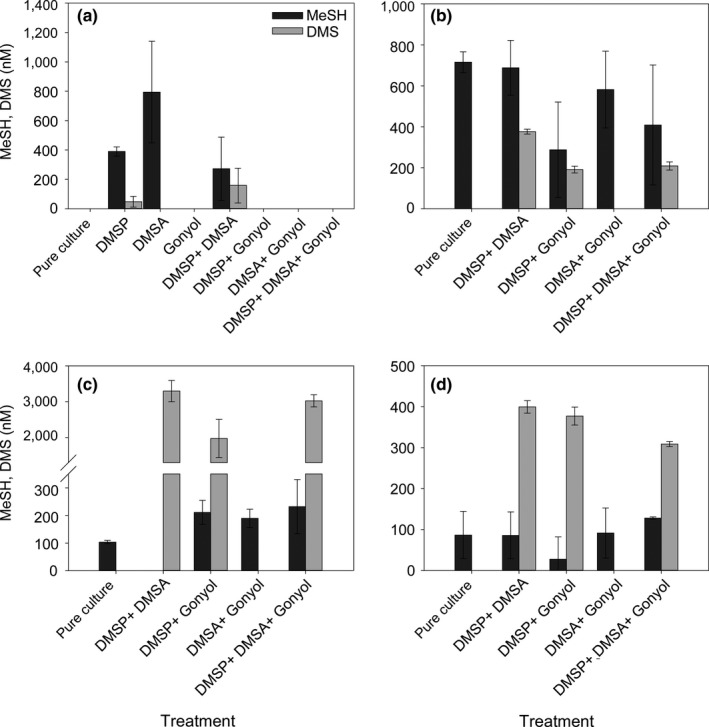
Mean substrate concentrations and net accumulation of volatile sulfur compounds methanethiol (MeSH) and dimethylsulfide (DMS) in combined substrate treatments with *R. pomeroyi* DSS‐3 (a, note the data for DMSP and DMS are taken from Figure 2 for comparison), *Sulfitobacter *sp*.* EE‐36 (b), *A. faecalis* M3A (c) and *Halomonas *sp. HTNK1 (d) after 24 hr incubation. Error bars represent the standard deviation between biological replicates, *n* = 4. Statistical evaluation is given in Table 3

The experiments described above indicate that DMSA (Figure 2g) and gonyol interfere with the release of volatile sulfur metabolites (Figure 2f). To explore the inhibitory action in a systematic manner, we added combinations of DMSP, DMSA, and gonyol to the bacteria and monitored the production of volatiles. Compared to the controls (Figure 2), we observed species‐specific inhibitory effects of DMSA and gonyol on the enzymes involved in DMSP metabolism. These effects manifested in a modulation of the release of volatile sulfur compounds. Addition of an equimolar DMSP/DMSA mixture (both at 3.3 µM) to *R. pomeroyi* indicated an antagonistic effect of DMSA on the net release of MeSH (272 ± 215 nM; Figure 3a) compared with 1,180 ± 350 nM as the sum of the single treatments (Figure 2e). Gonyol addition in all administered substrate combinations resulted in suppressed MeSH and DMS production from DMSP in *R. pomeroyi* (Figure 3a), which is in agreement with the findings on the effect of gonyol as single substrate (Figure 2e). The inhibitory action of gonyol on the MeSH production in *Sulfitobacter *sp. which was observed in the single substrate treatments (Figure 2f) could also be observed in the mixed substrate experiments (Figure 3b). The fact that there were significant concentrations of MeSH in treatments where gonyol was present in combination with at least one other osmolyte (DMSP, DMSA) might indicate a protective effect of DMSP and DMSA on the inhibitory influence of gonyol (compare Figures 2f and 3b). No inhibitory effect of gonyol on MeSH net release was observed in *A. faecalis* and *Halomonas *sp. (Figure 3c, d). The release of MeSH in *A. faecalis* (Figure 3c) likely resulted from the metabolization of gonyol which is consistent with the results from single substrate treatments (Figure 2g).

*Alcaligenes faecalis* with all three osmolytes DMSP, DMSA, and gonyol showed significantly higher net DMS production with 3,020 ± 170 nM than in the treatment with DMSP and gonyol (1970 ± 530; *p* = .009) and nearly the same as in the treatment containing DMSP or DMSA only (*p* = .134, Figure 3c). Together with the result that DMSA is not a source for DMS (Figure 2g), this suggests a protective effect of DMSA on the DMS‐production activity in *A. faecalis*.

*Halomonas *sp*.* showed a slightly different pattern than the other bacteria (Figure 3d). The addition of gonyol significantly affected DMS release in the DMSP/DMSA treatment (400 ± 15 nM) compared with DMSP/DMSA/gonyol (309 ± 6 nM; *p* < .001). Treatments of DMSP/gonyol (377 ±22 nM) and DMSP/DMSA were not significantly different (*p* = .144). This pattern could be caused by different types of DMSP‐catabolizing enzymes in this bacterium. The DddD enzyme from *Halomonas *sp. leads to 3‐hydroxypropionate as a by‐product (Todd et al., 2010, 2007), whereas all other DMS‐producing enzymes of the bacteria tested here co‐produce acrylate (Figure 1) (Curson, Sullivan, et al., 2011).

Taken together these results show that the phytoplankton‐derived zwitterionic dimethylsulfonio compounds DMSA and gonyol can affect the release of MeSH and the climatically active DMS by marine bacteria. Given their wide distribution (Table 2) implications of these findings for the marine sulfur cycle will have to be addressed using natural plankton communities. The additional sources for sulfur‐containing volatiles have to be considered as well as possible inhibitory effects that might serve as indirect regulators of the marine sources of volatile sulfur.

## 4 CONCLUSION

We show that the zwitterionic algal osmolytes DMSA and gonyol are widely distributed in phytoplankton. As a consequence, bacterial communities will often be exposed to mixtures of these structurally related dimethylsulfonio‐metabolites. The compounds and their inhibitory effect on the bacterial sulfur metabolism were highly species‐specific. All bacteria tested were capable of metabolizing these substrates. However, the involved pathways apparently differed. The enzymatic release of MeSH from DMSA suggests a so far unrecognized demethylation/demethiolation pathway. Furthermore, gonyol strongly interfered with volatile release from DMSP in *R. pomeroyi*. This suggests that gonyol affects the marine sulfur cycle by modulating the metabolization of other potential substrates including DMSP. Future studies should consider the differential effects of these molecules on purified enzymes as well as in complex plankton samples to further our understanding of the mechanisms in bacterial degradation of DMSP and related substances.

## CONFLICT OF INTEREST

None declared.

## AUTHOR CONTRIBUTION

Björn Gebser: Conceptualization; Formal analysis; Investigation; Methodology; Writing‐original draft; Writing‐review & editing. Kathleen Thume: Formal analysis; Investigation; Methodology; Validation; Writing‐review & editing. Michael Steinke: Conceptualization; Investigation; Methodology; Supervision; Writing‐review & editing. Georg Pohnert: Conceptualization; Resources; Writing‐original draft; Writing‐review & editing. 

## ETHICS STATEMENT

None required.

## Data Availability

All data are provided in the Results section and the Appendix of this manuscript.

## APPENDIX 1

### Synthetic procedures

#### DMSP and D6‐DMSP (as hydrochloride)

Dimethylsulfoniopropionate and D6‐DMSP were synthesized according to Chambers (Chambers, Kunin, Miller, & Hamada, 1987) by passing gaseous hydrogen chloride through a solution of anhydrous acrylic acid (Fluka) and dimethyl sulfide (Sigma‐Aldrich) or D6‐dimethyl sulfide (Sigma‐Aldrich), respectively. Dichloromethane was used as a solvent. Recrystallization of the resulting white solid from methanol/diethyl ether (MeOH/Et2O) gave DMSP and D6‐DMSP, respectively, as white needles.

### DMSP

^1^H‐NMR (400 MHz, CD3OD) δ ppm: 2.97–3.01 (m, 6 H/2 H, CH3/CH2), 3.54–3.57 (t, 2H, CH2, 3*J*HH = 6.87 Hz); 13C‐NMR (101 MHz, CD3OD) δ ppm: 26.45, 29.81, 40.83, 173.56.

### D6‐DMSP

^1^H‐NMR (400 MHz, CD3OD) δ ppm: 2.96–2.99 (t, 2H, CH2, 3*J*HH = 6.72 Hz), 3.52–3.55 (t, 2H, CH2, 3*J*HH =

642 6.72 Hz); 13C‐NMR (101 MHz, CD3OD) δ ppm: 26.00, 30.05, 40.79, 173.79.

#### DMSA and D6‐DMSA (as hydrobromide)

Dimethylsulfide‐Ac and D6‐DMS‐Ac were synthesized according to Howard (Howard & Russell, 1997) by addition of dimethyl sulfide (Sigma‐Aldrich) and D6‐dimethyl sulfide (Sigma‐Aldrich), respectively, to a well‐stirred solution of bromoacetic acid (Fluka) in dichloromethane. Recrystallization was carried out from MeOH/Et2O.

### DMSA

^1^H‐NMR (200 MHz, CD3OD) δ ppm: 2.99 (s, 6H), 4.50 (s, 2H); 13C‐NMR (101 MHz, CD3OD) δ ppm:25.76, 46.90, 167.20.

### D6‐DMSA

^1^H‐NMR (200 MHz, CD3OD) δ ppm: 4.49 (s, 2H); 13C‐NMR (101 MHz, CD3OD) δ ppm: 25.74, 46.88, 167.38.

#### Rac‐gonyol and rac‐D3‐gonyol (as hydroiodide)

Synthesis of racemic gonyol and D3‐gonyol was carried out according to Gebser (Gebser & Pohnert, 2013). Methylation of the thioether group of 3‐hydroxy‐5‐methylthiopentanoic acid in the final step was carried out in acetone using iodomethane (Sigma‐Aldrich) and D3‐iodomethane (Sigma‐Aldrich), respectively, as the alkylating agent. Reprecipitation of the raw product from MeOH/Et2O gave gonyol and D3‐gonyol, respectively, as yellow, partially crystalline oil.

### Gonyol

^1^H‐NMR (400 MHz, CD3OD) δ ppm: 1.89 – 2.01 (m, 1 H, CH2), 2.07 – 2.17 (m, 1 H, CH2), 2.47 – 2.60 (m, 2 H, CH2), 2.94 (s, 6 H, CH3), 3.44 – 3.54 (m, 2 H, CH2), 4.10 – 4.19 (m, 1 H, CH); 13C‐NMR (101 MHz, CD3OD) δ ppm: 25.62, 25.97, 31.68, 42.03, 42.52, 67.53, 174.63.

### D3‐gonyol

^1^H‐NMR (400 MHz, CD3OD) δ ppm: 1.89–2.01 (1H, m), 2.07–2.17 (1H, m), 2.47–2.60 (2H, m) 2.94, 2.95 (3H, s), 3.37–3.53 (2H, m), 4.10–4.19 (1H, m); 13C‐NMR (101 MHz, CD3OD) δ ppm: 25.84, 26.19, 31.98, 42.40, 42.77, 67.83, 174.78.
